# Laterality disorder and its association with an axial hypotonia and body spatial integration impairments may involve slowness and attention disorder: A case series of patients

**DOI:** 10.1192/j.eurpsy.2022.1157

**Published:** 2022-09-01

**Authors:** L. Vaivre-Douret

**Affiliations:** 1 National Institute of Health and Medical Research (INSERM UMR 1018-CESP), University of Paris-Saclay, UVSQ, Faculty Of Medicine, Villejuif, France; 2 Faculty of Health, Université de Paris, Department Of Medicine, Paris, France; 3 Imagine Institute Necker hospital, Department Of Endocrinology, Paris, France; 4 University Institute of France, (iuf), Paris, France; 5 Necker hospital AP-HP .Centre, Child Psychiatry, Paris, France

**Keywords:** attention disorder, Laterality dominance, trunk hypotonia, body spatial integration impairments

## Abstract

**Introduction:**

Lateralization is a complex process that evolves during the development of the child leading to the organization of the functional dominance with a body side. However, laterality is poorly examined.

**Objectives:**

The aim of this study was to explore the features of patients with a laterality disorder evidenced by a non-dominance affirmed of the handedness.

**Methods:**

A retrospective review of 25 cases of patients (15 children of 8-9 years and 10 adults of 26-42 years old) presenting a laterality disorder. All patients were assessed with the standardized assessment of neuropsychomotor functions battery (NP-MOT).It enables physical assessment of muscular tone of limbs and axial tone (trunk), laterality (tonic, spontaneous gestural, psychosocial handedness, and usual with objects for hand, foot, and eye), body spatial integration (in relation to self, imitation, objects and map), auditory attention, and others motor functions.

**Results:**

The study findings revealed poor level of the dominant laterality for all the patients (< 2DS) regarding the proximal tonic laterality (elbows) and the psychosocial subtests of mimed gestures (tending to ambidexterity). In addition, it was found a hypotonic trunk and difficulties in body spatial integration and in asymmetrical bimanual tasks with slowness and failures, similar dexterity performance right/left.Strong correlations between all these features and the personal history match the difficulties to focus a long time an attention holding posture without to move, and associated to a fatigability.

**Conclusions:**

A deep standardized examination of the laterality and tone may explain some components of the behavior in relation with hemispheric dominance impairment.

**Disclosure:**

No significant relationships.

